# New Benzoxazine Secondary Metabolites from an Arctic Actinomycete

**DOI:** 10.3390/md12052526

**Published:** 2014-04-30

**Authors:** Kyuho Moon, Chan-Hong Ahn, Yoonho Shin, Tae Hyung Won, Keebeom Ko, Sang Kook Lee, Ki-Bong Oh, Jongheon Shin, Seung-Il Nam, Dong-Chan Oh

**Affiliations:** 1 Natural Products Research Institute, College of Pharmacy, Seoul National University, Seoul 151-742, Korea; E-Mails: wanabkyo@snu.ac.kr (K.M.); dicafree5@snu.ac.kr (Y.S.); wth123@snu.ac.kr (T.H.W.); gogiup0218@snu.ac.kr (K.K.); sklee61@snu.ac.kr (S.K.L.); shinj@snu.ac.kr (J.S.); 2 Department of Agricultural Biotechnology, College of Agriculture and Life Science, Seoul National University, Seoul 151-921, Korea; E-Mails: chanhong@snu.ac.kr (C.-H.A.); ohkibong@snu.ac.kr (K.-B.O.); 3 Arctic Research Centre, Korea Polar Research Institute, Incheon 406-840, Korea; E-Mail: sinam@kopri.re.kr

**Keywords:** Arctic, marine actinomycete, secondary metabolite, benzoxazine, C-1027, fijiolides, disaccharide, isocitrate lyase

## Abstract

Two new secondary metabolites, arcticoside (**1**) and C-1027 chromophore-V (**2**), were isolated along with C-1027 chromophore-III and fijiolides A and B (**3**–**5**) from a culture of an Arctic marine actinomycete *Streptomyces* strain. The chemical structures of **1** and **2** were elucidated through NMR, mass, UV, and IR spectroscopy. The hexose moieties in **1** were determined to be d-glucose from a combination of acid hydrolysis, derivatization, and gas chromatographic analyses. Arcticoside (**1**) and C-1027 chromophore-V (**2**), which have a benzoxazine ring, inhibited *Candida albicans* isocitrate lyase. Chromophore-V (**2**) exhibited significant cytotoxicity against breast carcinoma MDA-MB231 cells and colorectal carcinoma cells (line HCT-116), with IC_50_ values of 0.9 and 2.7 μM, respectively.

## 1. Introduction

**Figure 1:**
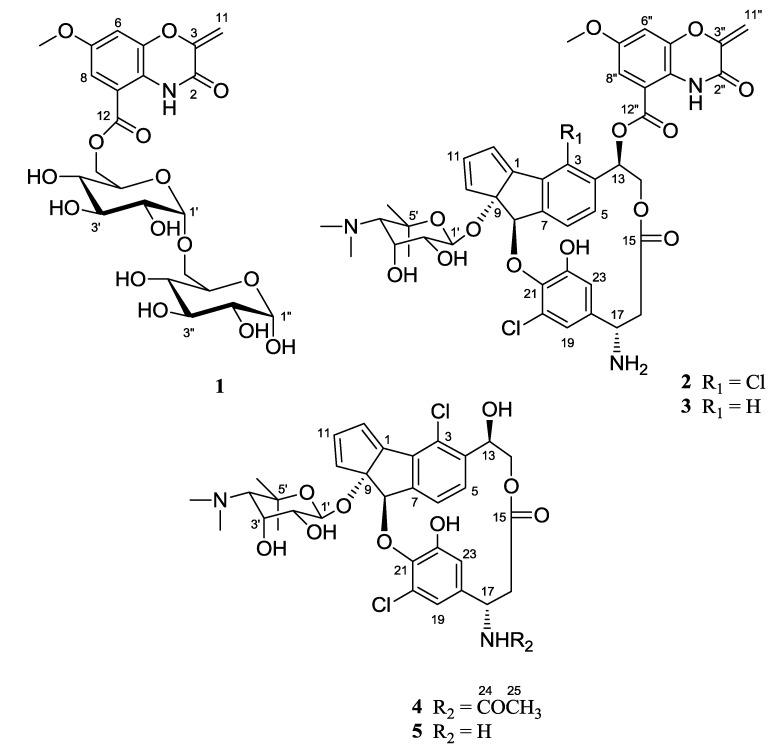
The structures of arcticoside (**1**), C-1027 chromophore-V (**2**), C-1027 chromophore-III (**3**), and fijiolides A and B (**4** and **5**).

Secondary metabolites produced by microorganisms are often developed into drugs; the success rate for the development of microbial products into drugs is 1.6%, whereas that for natural products and synthetic chemical compounds is 0.6% and 0.005%, respectively [1]. Although numerous microbial natural products were successfully marketed during the golden era of antibiotics between 1940 and 1960, an exhaustive investigation of readily accessible terrestrial microorganisms during the last two decades has resulted in the discovery of fewer bioactive compounds [2]. Therefore, microorganisms inhabiting relatively uninvestigated and extreme environments have attracted attention as new resources for bioactive compounds in drug discovery [3]. Such extreme environments include the deep sea, solfataric areas, abandoned mines, extremely saline environments and polar regions. For example, recent chemical studies of extremophiles have discovered ammonificins A and B from a marine hydrothermal vent bacterium [4], glionitrin A from a microbial population in an abandoned mine [5], and sungsanpin, a new lasso peptide from a deep-sea actinomycete [6]. Polar regions are exposed to extremely low temperatures. Consequently, polar regions are expected to harbor unique microbial communities that could biosynthesize novel bioactive compounds. However, only a few reports exist regarding the secondary metabolites of polar microorganisms [7,8]. As part of our continuing efforts to explore the chemical diversity of microorganisms from unique environments for drug discovery, we isolated actinomycete strains from the surface sediment taken at the East Siberian continental margin during the RV ARAON Arctic Expedition (ARA03B) in 2012. Then, we investigated the secondary metabolites of actinomycete strains by analyzing the chemical profiles of the bacteria by LC/MS. During the chemical screening of these secondary metabolites, it was found that the ART5 actinomycete strain, which was isolated from a sediment sample collected using the box core (ARA03B/26BOX-01, water depth = 354 m), produces compounds with distinctive UV spectra (λ_max_ 336–346 nm). Initial de-replication, based on our UV library interlinked with LC/MS, indicated that these compounds were likely unknown. Consequently, we scaled up the culture and isolated two new benzoxazine-bearing compounds, arcticoside (**1**) and C-1027 chromophore-V (**2**), and the previously reported compounds C-1027 chromophore-III (**3**) [9] and fijiolides A and B (**4** and **5**) [10]. Here, we report the isolation, structure determination, and biological activities of arcticoside (**1**) and C-1027 chromophore-V (**2**) (Figure 1).

## 2. Results and Discussion

### 2.1. Structural Elucidation

**Figure 2:**
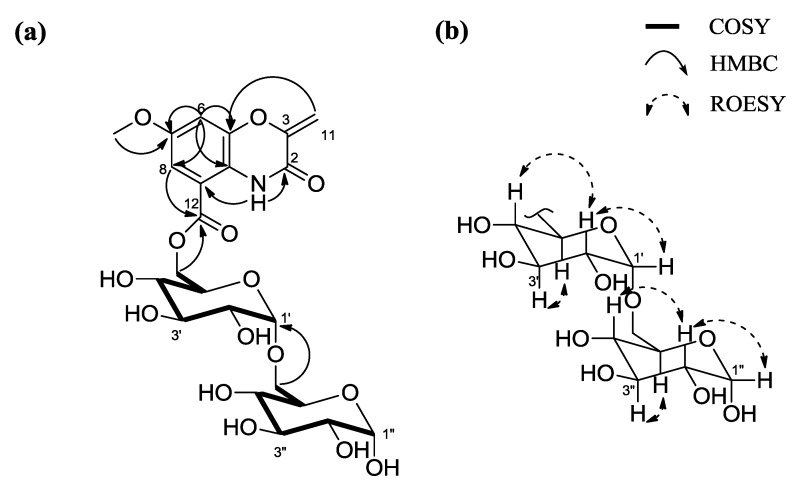
(**a**) Key ^1^H-^1^H COSY and HMBC correlations of **1**; (**b**) Relative configuration of the disaccharide of **1** based on the key ROESY correlations.

Arcticoside (**1**) was isolated as a pale yellow solid. The molecular formula of **1** was assigned as C_23_H_29_NO_15_ based on the ^1^H and ^13^C NMR data (Table 1) and HR-FAB mass data (*m*/*z* 559.1527 [M + H]^+^, calcd 559.1537 [M + H]^+^). The ^1^H NMR spectrum of **1** in DMSO-*d*_6_ displayed the signals from one amide proton (δ_H_ 10.11); *meta*-coupled aromatic protons [δ_H_ 7.13 (d, *J* = 2.5 Hz); 7.05 (d, *J* = 2.5 Hz)]; two olefinic protons (δ_H_ 5.48; 5.14); 14 protons attached to oxygen-bearing carbons, between 4.95 ppm and 3.14 ppm; and one methyl group (singlet at δ_H_ 3.76). The ^13^C NMR and HSQC spectra of **1** indicated the presence of two carbonyls (δ_C_ 165.3 and 154.5), eight carbons in the olefinic region between δ_C_ 154.3 and 98.7, 12 oxygen-bearing *sp*^3^ carbons between δ_C_ 93.7 and 60.6, and one methoxy carbon (δ_C_ 55.7). Detailed 1D and 2D NMR data analysis, including COSY, HSQC, HMBC, and TOCSY, were used to determine the structure of **1**. First, the COSY NMR data, the ^1^H-^1^H coupling of H-6 [δ_H_ 7.05 (d, *J* = 2.5)] and H-8 [δ_H_ 7.13 (d, *J* = 2.5)], and the long-range HMBC correlations between H-6 and C-5 (δ_C_ 142.2), C-7 (δ_C_ 154.3), C-8 (δ_C_ 108.6) and C-10 (δ_C_ 120.7) and between H-8 (δ_H_ 7.13) and C-6 (δ_C_ 107.2), C-7 (δ_C_ 154.3), C-9 (δ_C_ 114.5), C-10 (δ_C_ 120.7), and C-12 (δ_C_ 165.3), were used to establish the presence of a 6-membered aromatic ring. The oxazine moiety was inferred by long-range heteronuclear couplings from H_2_-11 (δ_H_ 5.48; 5.14) to C-2 (δ_C_ 154.5), C-3 (δ_C_ 147.4), and C-5 (δ_C_ 142.2) and by HMBC correlations from 1-NH (δ_H_ 10.11) to C-2 (δ_C_ 154.5), C-3 (δ_C_ 147.4), C-5 (δ_C_ 142.2), C-9 (δ_C_ 114.5), and C-10 (δ_C_ 120.7). In addition, the methoxy group was connected to C-7 based on the 3-bond HMBC of the methyl singlet protons (δ_H_ 3.76) with C-7 (δ_C_ 154.3). Further COSY, TOCSY, and HMBC analysis of the NMR data identified two partial hexose structures. The TOCSY NMR correlations clearly showed two spin systems from the anomeric proton H-1′ [δ_H_ 4.95 (δ_c_ 93.7)] to H_2_-6′ [δ_H_ 4.49 and 4.40 (δ_c_ 64.9)] and from the anomeric proton H-1″ [δ_H_ 4.91 (δ_c_ 93.7)] to H_2_-6″ [δ_H_ 3.54 and 3.47 (δ_c_ 60.6)]. The H_2_-6″–C-1′ three-bond coupling in the HMBC spectrum indicated a 1′-6″ glycosidic linkage between the two sugars. The disaccharide moiety was connected to the benzoxazine ring through the ester functional group at C-12, based on the HMBC correlation from H_2_-6′ to C-12, which completed the planar structure of arcticoside (**1**) (Figure 2).

**Table 1:** NMR data for arcticoside (**1**) in DMSO-*d_6_*.

No.	δ_H_^a^	Mult (*J* in Hz)	δ_C_^b^	
2			154.5	C
3			147.4	C
5			142.2	C
6	7.05	d (2.5)	107.2	CH
7			154.3	C
8	7.13	d (2.5)	108.6	CH
9			114.5	C
10			120.7	C
11a 11b	5.48 5.14	d (2.0) d (2.0)	98.7	CH_2_
12			165.3	C
1′	4.95	d (3.5)	93.7	CH
2′	3.35	dd (9.5, 3.5)	71.3	CH
3′	3.58	dd (9.5, 9.5)	72.8	CH
4′	3.20	dd (9.5, 9.5)	70.2	CH
5′	4.13	ddd (9.5, 5.5, 2.0)	69.2	CH
6′a 6′b	4.49 4.40	dd (11.5, 2.0) dd (11.5, 5.5)	64.9	CH_2_
1″	4.91	d (3.5)	93.7	CH
2″	3.23	dd (9.5, 3.5)	71.6	CH
3″	3.56	dd (9.5, 9.5)	72.7	CH
4″	3.14	dd (9.5, 9.5)	70.0	CH
5″	3.67	ddd (9.5, 4.5, 2.0)	72.6	CH
6″a 6″b	3.54 3.47	dd (12.0, 2.0) dd (12.0, 4.5)	60.6	CH_2_
1-NH	10.11	s		
7-OMe	3.76	s	55.7	CH_3_

^a^ 600 MHz; ^b^ 125 MHz.

The relative configurations of the two sugars were established by analyzing the one-bond C-H coupling constants of the anomeric protons and carbons, the ^1^H-^1^H vicinal coupling constants, and the ROESY NMR spectrum. The magnitude of ^1^*J*_CH_ (171 Hz) between C-1′ and H-1′ was clearly assigned the α-configuration [11], placing the anomeric proton (H-1′) at the equatorial position. The large couplings (*J*_H2′3′_ = 9.5 Hz, *J*_H3′4′_ = 9.5 Hz, *J*_H4′5′_ = 9.5 Hz) indicated that H-3′, H-4′, and H-5′ were in the axial position. The strong ROESY correlation between H-2′ and H-4′ established the axial position of H-2′. Based on the relative configuration, this hexose was determined to be glucose. The ^1^*J*_CH_ of the anomeric proton and carbon and ^1^H-^1^H coupling constants of H-2″-H-5″ were identical to those for the first sugar, which indicated that the other hexose was also glucose. For the absolute configurations of the two glucose moieties, arcticoside (**1**) was subjected to acid hydrolysis. Then, the hydrolysate was derivatized with hexamethyldisilazane and TMSCl. The products were analyzed using gas chromatography, which indicated that both of these glucose moieties possessed the d-configuration (see Supplementary Information, Figure S15).

To the best of our knowledge, **1** is structurally novel, as no benzoxazine disaccharide has been previously reported. A few benzoxazine monosaccharides have been reported from a mangrove plant, *Acanthus illicifolius* [12]. However, these benzoxazine compounds possess a glycosidic linkage at C-3 instead of an exomethylene group (C-11). In addition, the substituents of the benzoxazine in **1**, such as the methoxy and ester functional groups, differ from the substituents in the plant-derived compounds. The identical benzoxazine ring system was isolated as a degradation product of C-1027 chromophore [13,14]. However, our discovery of this glycosylated benzoxazine compound may indicate that this benzoxazine ring can independently serve as a substrate for biosynthetically distinct natural products in addition to the C-1027 chromophore derivatives.

C-1027 chromophore-V (**2**) was isolated as a colorless noncrystalline solid. The molecular formula of **2** was determined to be C_43_H_43_Cl_2_N_3_O_13_ from the HR-FAB mass data (*m*/*z* 880.2259 [M + H]^+^, calcd 880.2251 [M + H]^+^) and ^1^H and ^13^C NMR data (Table 2). Before analyzing the structure of **2** with the 1D and 2D NMR data, we hypothesized that compound **2** was a C-1027 derivative because the UV spectra of compounds **2** and C-1027 chromophore-III (**3**) were similar. The chemical structure of **3 **was confirmed by comparing its NMR and physiochemical data with literature data [9]. Moreover, the molecular ion of **2** is 34 daltons heavier than that of **3**. In addition, the isotopic pattern of the ions showed a 100:65:10 ratio of [M + H]^+^:[M + 2 + H]^+^:[M + 4 + H]^+^, which indicated an additional chlorine substituent in **2** instead of a hydrogen.

The detailed structure of **2** was determined from the 1D and 2D NMR experimental data, including COSY, HSQC, HMBC, and ROESY. The coupling constants and COSY correlations of the double bond protons, including H-10 [δ_H_ 6.73 (d, *J* = 5.0 Hz)], H-11 [δ_H_ 6.74 (dd, *J* = 5.0, 1.5 Hz), and H-12 [δ_H_ 6.85 (d, *J* = 1.5 Hz)], indicated that these three protons are part of a cyclopentadiene system [15]. The COSY and HMBC correlations of the two *ortho*-coupled aromatic protons of H-5 [δ_H_ 7.03 (d, *J* = 8.0 Hz)] and H-6 [δ_H_ 7.33 (d, *J* = 8.0 Hz)] indicated a 1,2,3,4-tetrasubstituted benzene ring. These two cyclic sub-structures were connected to a cyclopenta[*a*]indene system according to the long-range HMBC correlations from H-6 (δ_H_ 7.33) to C-2 (δ_C_ 134.2) and C-8 (δ_C_ 83.5); from H-8 (δ_H_ 6.02) to C-1 (δ_C_ 148.8), C-2 (δ_C_ 134.2), C-6 (δ_C_ 125.9), C-7 (δ_C_ 149.0), and C-9 (δ_C_ 99.7); and from H-12 (δ_H_ 6.85) to C-1 (δ_C_ 148.8), C-2 (δ_C_ 134.2), C-9 (δ_C_ 99.7), and C-10 (δ_C_ 135.6). The H-13 oxygenated methine proton (δ_H_ 6.01), which was coupled with the H_2_-14 oxygenated methylene protons (δ_H_ 4.57; 4.16), also displayed long-range HMBC correlations to carbons C-3 (δ_C_ 126.1), C-4 (δ_C_ 136.1), and C-5 (δ_C_ 130.0), which showed that it was a C-4 substituent on the cyclopenta[*a*]indene. The structure 3′-chloro-5′-hydroxy-β-tyrosine moiety was inferred from the COSY and HMBC NMR spectra. First, the H-17 methine proton (δ_H_ 4.23) showed a correlation with the H_2_-16 methylene protons (δ_H_ 3.01; 2.36) in the COSY. Second, the long-range HMBC correlations from H-19 (δ_H_ 6.87) to C-17 (δ_C_ 51.2), C-20 (δ_C_ 129.6), C-21 (δ_C_ 139.7), and C-23 (δ_C_ 114.6); from H-23 (δ_H_ 6.17) to C-17 (δ_C_ 51.2), C-19 (δ_C_ 114.3), C-21 (δ_C_ 139.7), and C-22 (δ_C_ 152.4); and from the 22-OH phenol proton (δ_H_ 8.56) to C-21 (δ_C_ 139.7) and C-22 (δ_C_ 152.4) supports the presence of a 3′-chloro-5′-hydroxy-β-tyrosine moiety. The benzoxazine partial structure was assigned in a similar manner to that for **1**. Sequential structural assignment of the amino sugar was based on the COSY and HMBC analyses of the NMR spectra. COSY correlations from the anomeric proton H-1′ [δ_H_ 4.54 (d, *J* = 8.0 Hz)] to H-4′ [δ_H_ 3.16 (br s.)] elucidated the spin system of the amino sugar. In the HMBC spectrum, the *N*,*N*-dimethyl proton (δ_H_ 2.86) was correlated with C-4′ (δ_C_ 69.2), two methyl groups (δ_H_ 1.44; 1.46), C-5′ (δ_C_ 74.9) and C-4′ (δ_C_ 69.2), which completed the amino sugar substructure.

**Table 2:** NMR data for the C-1027 chromophore-V (**2**) in DMSO-*d*_6_.

No.	δ_H_^a^	Mult (*J* in Hz)	δ_C_^b^	
1			148.8	C
2			134.2	C
3			126.1	C
4			136.1	C
5	7.03	d (8.0)	130.0	CH
6	7.33	d (8.0)	125.9	CH
7			149.0	C
8	6.02	s	83.5	CH
9			99.7	C
10	6.73	d (5.0)	135.6	CH
11	6.74	dd (5.0, 1.5)	138.5	CH
12	6.85	d (1.5)	129.9	CH
13	6.01	dd (10.5, 5)	74.0	CH
14a 14b	4.57 4.16	d (10.5, 10) d (10.5, 5)	62.8	CH_2_
15			167.9	C
16a 16b	3.01 2.36	d (12.5) d (12.5)	41.3	CH_2_
17	4.23	d (12.5)	51.2	CH
18			133.6	C
19	6.87	d (1.5)	114.3	CH
20			129.6	C
21			139.7	C
22			152.4	C
23	6.17	d (1.5)	114.6	CH
1′	4.54	d (8.0)	92.9	CH
2′	2.97	dd (8.0, 3.0)	69.3	CH
3′	4.09	br s	67.4	CH
4′	3.16	br s	69.2	CH
5′			74.9	C
2″			154.5	C
3″			147.3	C
5″			142.1	C
6″	7.12	d (2.5)	107.4	CH
7″			154.6	C
8″	7.29		109.1	CH
9″			113.5	C
10″			121.2	C
11″a 11″b	5.48 5.16	s s	98.9	CH_2_
12″			164.7	C
4′-NMe_2_ 4′-NMe_2_	2.86 2.86	br s br s	44.3 42.7	CH_3_ CH_3_
6′-Meα 6′-Meβ	1.44 1.46	s s	31.3 21.3	CH_3_ CH_3_
7″-OMe	3.79	s	55.8	CH_3_
17-NH_2_	9.08			
22-OH	8.56	s		
2′-OH	5.12			
3′-OH	5.49			CH
1″-NH	10.05	s		

^a^ 600 MHz; ^b^ 125 MHz.

**Figure 3:**
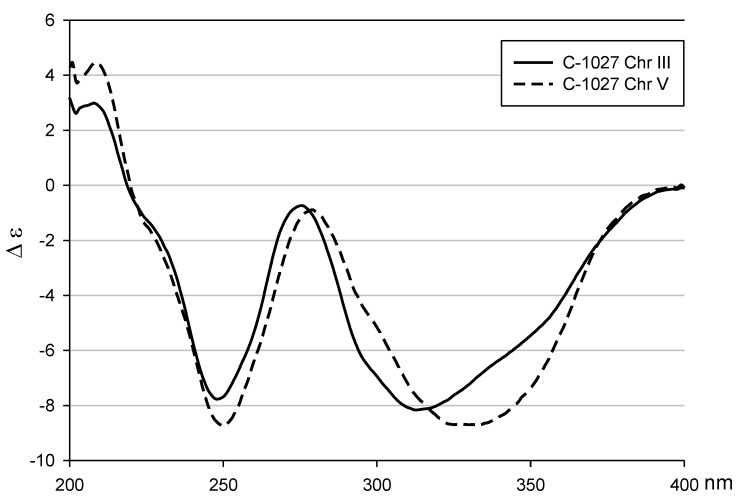
CD spectra of C-1027 chromophore-V (**2**) and C-1027 chromophore-III (**3**) in MeOH.

The four partial structures, the cyclopenta[*a*]indene, 3′-chloro-5′-hydroxy-β-tyrosine, benzoxazine, and amino sugar, were connected using the heteronuclear couplings from H-13 (δ_H_ 6.01) to the C-12″ carbons (δ_C_ 164.7), which is part of the benzoxazine structure. A three-bond HMBC correlation from H-8 (δ_H_ 6.02) to C-21 (δ_C_ 139.7) indicated a connection between C-8 and C-21 through an oxygen atom. The anomeric proton H-1′ (δ_H_ 4.54) displayed an HMBC correlation to C-9 (δ_C_ 99.7), which suggested a linkage between the amino sugar and the cyclopenta[*a*]indene. The strong ROESY correlations among H-2′ (δ_H_ 2.97), H-3′ (δ_H_ 4.09), and H-4′ (δ_H_ 3.16) identified the amino sugar as 4-deoxy-4-dimethylamino-5,5-dimethyl-ribopyranose with an equatorial glycoside linkage. Finally, locating a chlorine atom at the open position of C-3 established the structure of **2**. The absolute configuration of **2** was deduced to be identical to that of **3** by comparing the circular dichroism (CD) spectra of **2** and **3** (Figure 3). Fijiolides A and B (**4** and **5**) were isolated with **1**–**3**. The structures of fijiolides A and B were identified by comparing their NMR and physiochemical data with the literature data [10]. Fijiolides A and B (**4** and **5**) were utilized to evaluate biological activity together with the benzoxazinated compounds (**1**–**3**).

The incorporation of a single chlorine atom into an aromatic ring via cycloaromatization of an enediyne precursor has been previously reported in cyanosporasides [16], sporolides [17], and fijiolides [10] from marine actinomycetes in seawater-based cultures. The mechanism of haloaromatization from an enediyne through a Bergman diradical intermediate is based on quenching one of the radicals with chloride in a halogen-rich environment before protonation [18]. The enediyne form of C-1027 chromophore [19] was detected in the organic extract of the bacterial culture by LC/MS. However, the purification of the enediyne form was not conducted because of its low quantity.

### 2.2. Bioactivity Results

#### 2.2.1. Inhibition of *Candida albicans* Isocitrate Lyase

Arcticoside (**1**) showed considerable inhibitory activity against *Candida albicans* isocitrate lyase (ICL), an enzyme that plays an important role in the pathogenicity of *C. albicans* [20], with an IC_50_ of 30.4 μM. In addition, C-1027 chromophore-V (**2**) and C-1027 chromophore-III (**3**) also displayed inhibitory activity (IC_50_ values of 37.9 μM and 25.6 μM, respectively) towards ICL. Interestingly, fijiolides A and B (**4** and **5**), which do not possess benzoxazine moieties, exhibited no activity even at high concentrations (100 μg/mL). These results support our assumption that benzoxazine could be a pharmacophore for the inhibition of ICL. However, the structure-activity relationships of the benzoxazine pharmacophore should be further evaluated.

#### 2.2.2. Evaluation of Anti-Proliferative Activity

The cytotoxicities of **1**–**3** were tested against the following human carcinoma cell lines: HCT116 (colon cancer), A549 (lung cancer), SNU638 (gastric cancer), SK-HEP1 (liver cancer), K562 (leukemia cancer), and MDA-MB231 (breast cancer). Arcticoside (**1**) did not display notable inhibitory activity. However, C-1027 chromophore-V (**2**) significantly inhibited MDA-MB231 and HCT116 with IC_50_ values of 0.9 and 2.7 μM, respectively. The cytotoxic effects of **2** against A549, SNU638, K562, and SK-HEP1 were moderate (IC_50_ = 14.7, 9.8, 25.1, and 7.9 μM, respectively). In addition, C-1027 chromophore-III (**3**) showed cytotoxicity against all of the tested cell lines, with IC_50_ values of 0.6, 2.2, 1.1, 0.9, 4.8, and 44.0 μM for the HCT116, A549, SNU638, SK-HEP1, K562, and MDA-MB231 cell lines, respectively.

## 3. Experimental Section

### 3.1. General Experimental Procedures

Optical rotations were measured using a JASCO P-200 polarimeter (sodium light source, JASCO, Easton, PA, USA), and UV spectra were obtained with a Perkin Elmer Lambda 35 UV/VIS spectrometer (Perkin Elmer, Waltham, MA, USA). IR spectra were obtained with a Thermo NICOLET iS10 spectrometer (Thermo, Madison, CT, USA). Low-resolution electrospray ionization source mass spectra were acquired with an Agilent Technologies 6130 quadrupole mass spectrometer coupled to an Agilent Technologies 1200 series HPLC (Agilent Technologies, Santa Clara, CA, USA). High-resolution mass spectra (HRMS) were obtained using a Jeol JMS-700 FAB spectrometer (Jeol, München, Germany) at the National Center for Inter-university Research Facilities at Seoul National University. ^1^H, ^13^C, and 2D NMR spectra were obtained using a Bruker Avance 600 MHz spectrometer (Bruker, Billerica, MA, USA) at the National Center for Inter-university Research Facilities. NMR experiments were conducted at 25 °C and 6 mg of arcticoside (**1**) and 7 mg of C-1027 chromophore-V (**2**) were dissolved in 600 μL of DMSO-*d*_6_ for NMR samples, respectively (17.9 mM for **1**; 13.3 mM for **2**). CD spectra were obtained with an Applied Photophysics Chirascan with a circular dichroism detector (Applied Photophysics, Leatherhead Surrey, UK) at the NICEM (National Instrumentation Center for Environmental Management at College of Agriculture & Life Sciences, Seoul National University).

### 3.2. Bacterial Material, Cultivation and Extraction

During the RV ARAON Arctic Expedition in 2012, a surface sediment sample was collected in the East Siberian continental margin (75° 22.2743′ N, 177° 17.4846′ E) of Arctic Ocean by box coring. A portion of the sample (1 g) was dried, diluted in 12 mL of sterilized artificial seawater (1/3 dilution), and vortexed. The mixture was spread on Actinomycete Isolation medium (1 L of seawater, 18 g of agar, 20 mg/L cycloheximide, 20 mg/L nystatin, and 10 mg/L nalidixic acid), A5 (750 mL of seawater, 250 mL of distilled water, 18 g of agar, 40 mg/L cycloheximide, and 25 mg/L nalidixic acid), A6 (1 L of seawater, 18 g of agar, and 5 mg/L polymyxin B sulfate), and A7 (1 L of seawater, 18 g of agar, and 5 mg/L kanamycin) media. The plates were incubated at 20 °C for one month. Strain ART5 was isolated from the Actinomycete Isolation medium. Sequence analysis of 16S rDNA revealed that strain ART5 is most similar to *Streptomyces* *sundarbansensis* (99% identity).

Strain ART5 was cultivated in 1 L of YEME medium (4 g of yeast extract, 10 g of malt extract, 4 g of glucose, and 27 g of artificial sea salt). A total of 60 L of bacterial liquid culture was prepared. After 5 days on a rotary shaker at 180 rpm and 30 °C, Amberlite XAD-7 resin (Sigma-Aldrich, St. Louis, MO, USA) was added to the culture medium (20 g/L) before shaking the culture and resin at 180 rpm for 3 h. The resin was recovered using cheesecloth before eluting with acetone. The acetone extract was concentrated *in vacuo* to yield 25 g of dry material.

### 3.3. Isolation of Arcticoside (1) and C-1027 Chromophore-V (2)

One third of the crude extract was fractionated with 150 mL each of 20%, 40%, 60%, 80%, and 100% MeOH in water using a packed C_18_ (20 g) column. Arcticoside (**1**) and C-1027 chromophore-V (**2**) were found in the 40% and 60% and the 80% and 100% fractions, respectively. Compound **1** was obtained at a retention time of 23 min by semi-preparative reversed-phase HPLC (Kromasil 100-5-C_18_ 250 × 10 mm, flow rate 2 mL/min, UV 230 nm detection, and 22.5% aqueous acetonitrile isocratic solvent (0.1% formic acid)). Compound (**2**) was obtained at 45 min by HPLC with the same C_18_ column (flow rate 2 mL/min, UV 360 nm detection, and gradient 10%–60% aqueous acetonitrile (0.1% TFA) over 70 min). Finally, the procedure was repeated three times to afford purified arcticoside (**1**) (18 mg) and chromophore-V (**2**) (7 mg).

#### 3.3.1. Arcticoside (**1**)

Amorphous pale yellow solid; 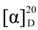 +19 (c 0.1 in MeOH); IR (KBr) 3388, 2965, 2938, 1680, 1615, 1507, 1259, 1053, 1032 cm^−1^; UV (MeOH) λ_max_ (log ε) 346 (0.29), 220 (1.28), 209 (1.31) nm; ^1^H and ^13^C NMR (see Table 1); and HRFABMS [M + H]^+^ *m*/*z* 559.1527 (calcd for C_23_H_29_NO_15_, 559.1537).

#### 3.3.2. C-1027 Chromophore-V (**2**)

Colorless noncrystalline solid; 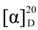 −412 (c 0.2 in MeOH); IR (KBr) 3428, 2933, 1683, 1438, 1366, 1207, 1135, 1051, 1028, 1007, 841 cm^−1^; UV (MeOH) λ_max_ (log ε) 336 (0.19), 210 (1.42) 206 (1.43) nm; ^1^H and ^13^C NMR (see Table 2); and HRFABMS [M + H]^+^ *m*/*z* 880.2259 (calcd for C_43_H_44_^35^Cl_2_N_3_O_13_, 880.2251).

### 3.4. Acid Hydrolysis and GC Analysis of Arcticoside (1)

A solution of **1** (2.0 mg) in 3 N HCl (1.0 mL) was stirred at 80 °C. The reaction mixture was cooled to room temperature over 5 h and concentrated under a stream of N_2_. The residue was dissolved in pyridine (1.0 mL). Then, hexamethyldisilazane (HMDS) and TMSCl (60 μL, v/v =2:1) were added, to the solution at 60 °C during 1 h. The solvent was removed, and the reaction mixture was partitioned into CH_2_Cl_2_ and H_2_O (2 mL, v/v = 1:1). The organic phase was analyzed by GC (HP5 column; 0.32 mm × 30 m) with the injector and detector held at 200 °C. After injection, the temperature of the GC oven was maintained at 60 °C for 3 min before increasing to 200 °C at 4 °C/min. Finally, the temperature was maintained at 200 °C for 3 min. The most intense peak of the reaction mixture had a retention time of 32.75 min. The retention times for authentic samples of d-glucose and l-glucose, after being analyzed in the same manner before each injection, were 32.74 and 32.91 min, respectively. Co-injection of the silylated d-glucose standard with the hydrolysate gave a single peak at 32.77 min.

### 3.5. Inhibition of the Isocitrate Lyase (ICL) Activity Assay

The formation of glyoxylate phenylhydrazone in the presence of phenylhydrazine and isocitrate was measured spectrophotometrically at 324 nm. The enzyme reaction mixture (1 mL) included 1.27 mM threo-DS (+) isocitrate, 3.75 mM MgCl_2_, 4.1 mM phenylhydrazine, 20 mM sodium phosphate buffer (pH 7.0), and 2.5 μg/mL purified *C. albicans* ICL. The reaction was performed at 37 °C for 30 min, and the protein concentration was measured using the Bradford method and a Bio-Rad protein assay Kit (Bio-Rad, Alfred Nobel Dr. Hercules, CA, USA) with bovine serum albumin as a standard. A positive control of 3-nitropropionic acid was used, and it inhibited ICL with an IC_50_ value of 35.2 μM.

### 3.6. Anti-Proliferative Activity Assay

Cell proliferation was analyzed using the sulforhodamine B (SRB) assay. Six human cancer cell lines (HCT116, MDA-MB231, SNU638, A549, K562, and SK-HEP1 at 3 × 10^4^ cells/mL) were seeded in 96-well plates at different compound concentrations before incubating at 37 °C in a humidified atmosphere with 5% CO_2_. After 72 h of treatment, the cells were fixed with a 10% TCA solution for 1 h before staining the cellular proteins with 0.4% SRB in 1% acetic acid. The stained cells were dissolved in 10 mM Tris buffer (pH 10.0). The effects of the compounds on cell proliferation were calculated as percentages relative to the solvent-treated control. In addition, the IC_50_ values were determined by nonlinear regression analysis (percent survival *versus* concentration).

## 4. Conclusions

Our study of bioactive secondary metabolites from Arctic actinomycete strain ART5, *Streptomyces* sp., resulted in the discovery of the new benzoxazine-bearing compounds arcticoside (**1**) and C-1027 chromophore-V (**2**), which showed inhibitory activity against *Candida albicans* isocitrate lyase. The structure of arcticoside (**1**) is intriguing because it indicates that the benzoxazine ring, which was previously identified to be a part of the C-1027 chromophore derivatives, is utilized through a different biosynthetic pathway. An additional chlorine substituent on **2** (compared to the other C-1027 derivatives) most likely originated from seawater-based culture because the bacterial strain was isolated from a sample of Arctic Ocean sediment. The preliminary structure-activity relationship study with fijiolides A and B (**4** and **5**), which do not include a benzoxazine ring, indicated that the benzoxazine ring may be essential for activity against isocitrate lyase. Overall, the discovery of these new bioactive secondary metabolites from a bacterium that inhabits sediment in the Arctic Ocean provides evidence that chemical investigations of microorganisms in relatively uninvestigated polar regions may result in significant natural chemical diversity for drug discovery.

## Supplementary Files

Supplementary File 1 Supplementary Information (PDF, 560 KB)
