# Supplementary material for: New Benzoxazine Secondary Metabolites from an Arctic Actinomycete
Source: Mar Drugs. 2014 Apr 30;12(5):2526–38. doi: 10.3390/md12052526 (PMC4052304; doi:10.3390/md12052526)
Supplement: Supplementary File 1 — Supplementary Information (PDF, 560 KB) [file marinedrugs-12-02526-s001.pdf]

Supplementary Information

Figure S1. ^{1}H NMR spectrum (600 MHz) of Arcticoside (**1**) in DMSO-d_6.
Figure S2. ^{13}C NMR spectrum (150 MHz) of Arcticoside (**1**) in DMSO-d_6.
Figure S3. COSY spectrum (600 MHz) of Arcticoside (**1**) in DMSO-d_6.
Figure S4. HSQC spectrum (600 MHz) of Arcticoside (**1**) in DMSO-d_6.
Figure S5. HMBC spectrum (600 MHz) of Arcticoside (**1**) in DMSO-d_6.
Figure S6. TOCSY spectrum (600 MHz) of Arcticoside (**1**) in DMSO-d_6.
Figure S7. ROESY spectrum (600 MHz) of Arcticoside (**1**) in DMSO-d_6.
Figure S8. ^{1}H NMR spectrum (600 MHz) of C-1027 Chr-V (**2**) in DMSO-d_6.
Figure S9. ^{13}C NMR spectrum (150 MHz) of C-1027 Chr-V (**2**) in DMSO-d_6.
Figure S10. COSY spectrum (600 MHz) of C-1027 Chr-V (**2**) in DMSO-d_6.
Figure S11. HSQC spectrum (600 MHz) of C-1027 Chr-V (**2**) in DMSO-d_6.
Figure S12. HMBC spectrum (600 MHz) of C-1027 Chr-V (**2**) in DMSO-d_6.
Figure S13. ROESY spectrum (600 MHz) of C-1027 Chr-V (**2**) in DMSO-d_6.
Figure S14. 16S rDNA sequence data of *Streptomyces* sp. ART 5.
Figure S15. (**a**) The GC chromatograms of the silylated arcticoside sugar. (**b**) Co-injection of the silylated D-glucose standard and the silylated arcticoside sugar. D-Glucose and the arcticoside sugar eluted at the same retention time. (**c**) Co-injection of the silylated L-glucose standard and the silylated arcticoside sugar. L-Glucose and the arcticoside sugar were separated.

Figure S1. ^{1}H NMR spectrum (600 MHz) of Arcticoside (**1**) in DMSO-d_6.

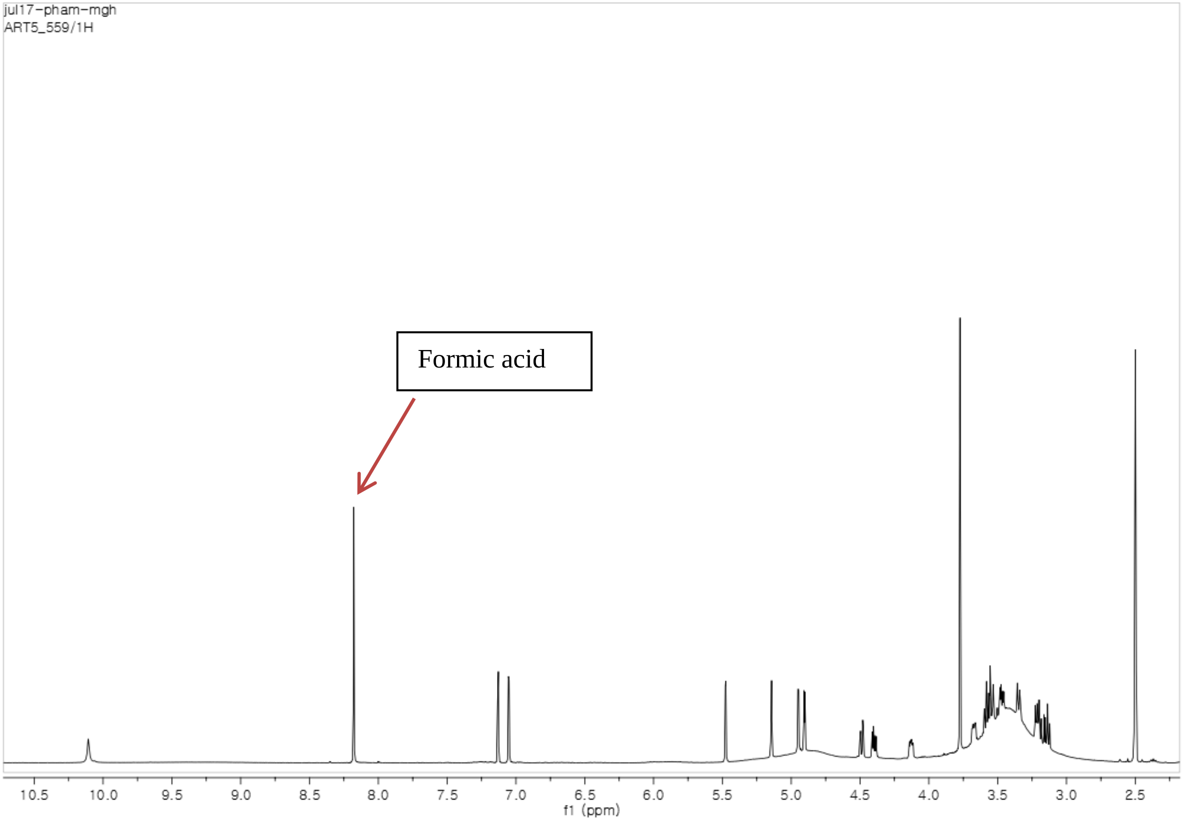

Figure S2. ^{13}C NMR spectrum (150 MHz) of Arcticoside (**1**) in DMSO-d_6.

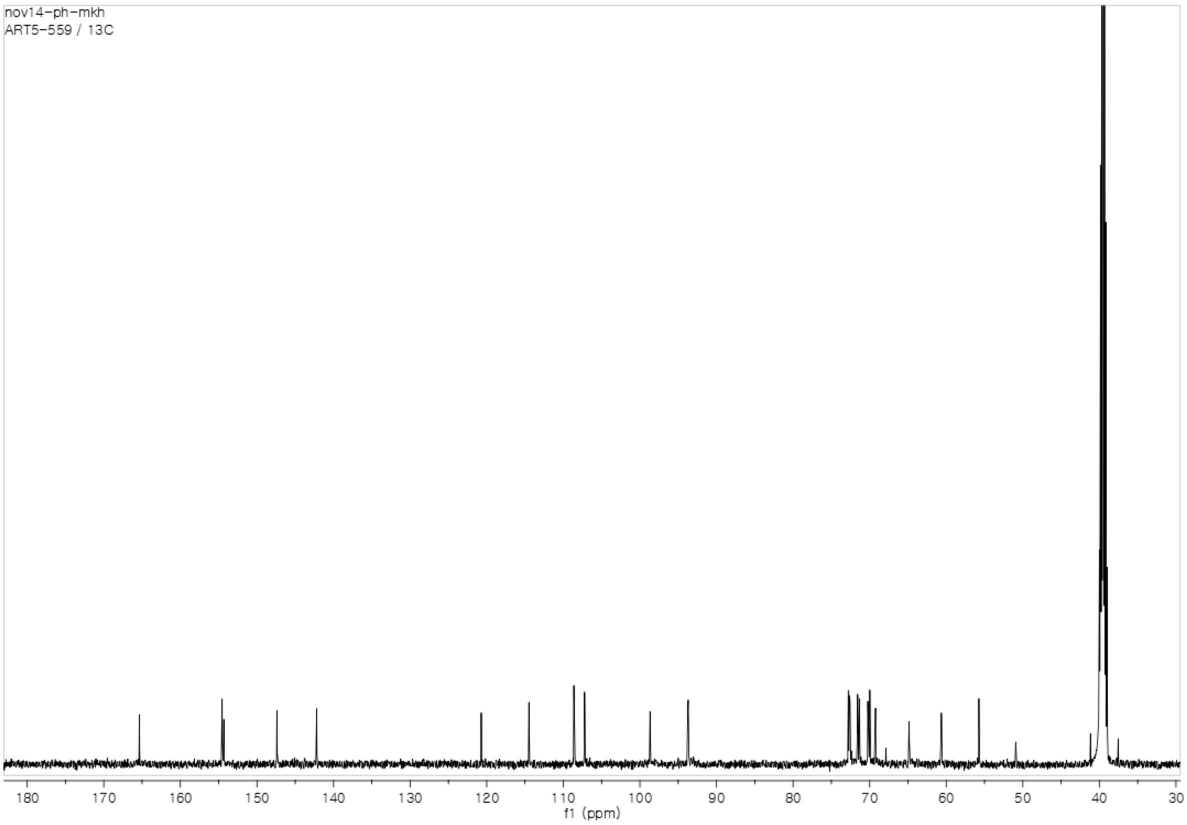

Figure S3. COSY spectrum (600 MHz) of Arcticoside (**1**) in DMSO-d_6.

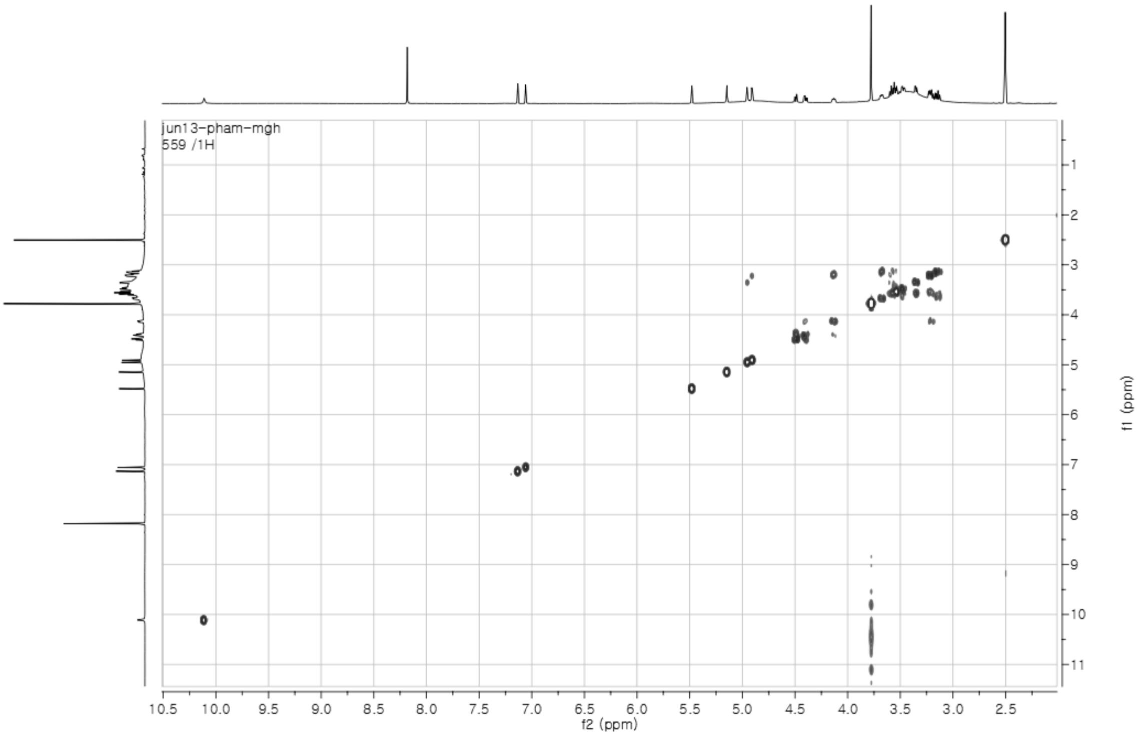

Figure S4. HSQC spectrum (600 MHz) of Arcticoside (**1**) in DMSO-d_6.

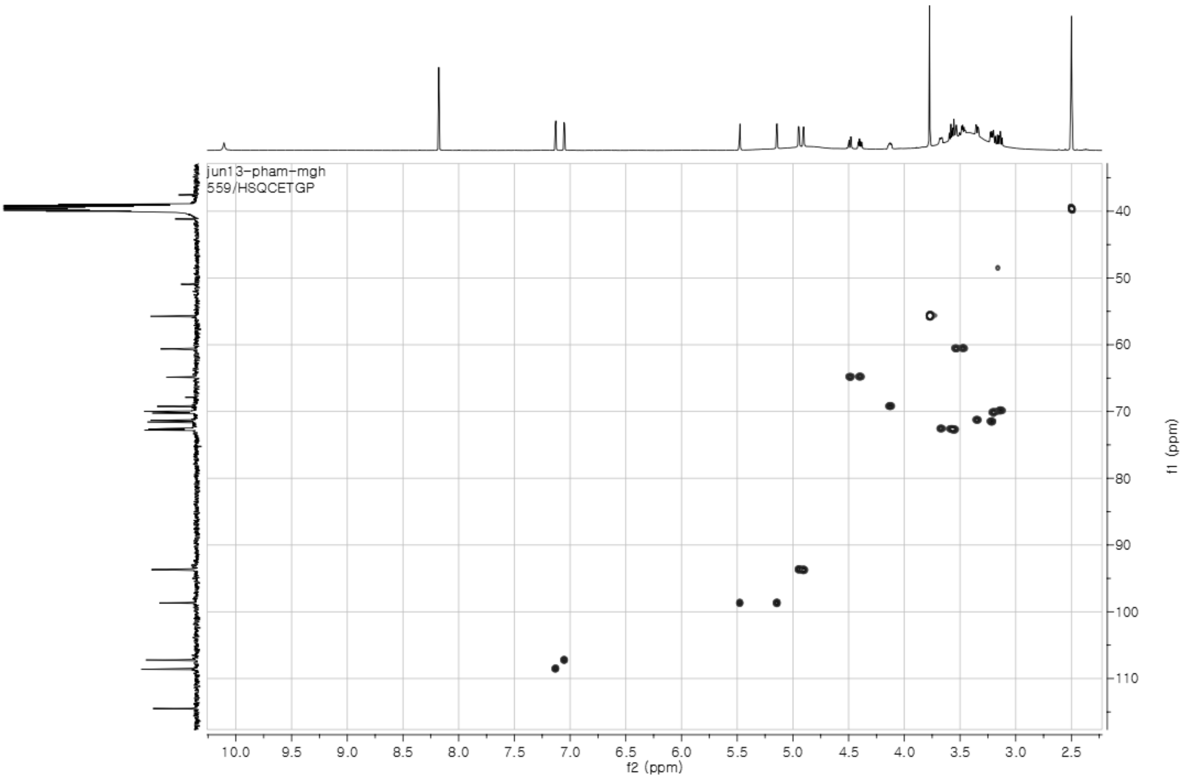

Figure S5. HMBC spectrum (600 MHz) of Arcticoside (**1**) in DMSO-d_6.

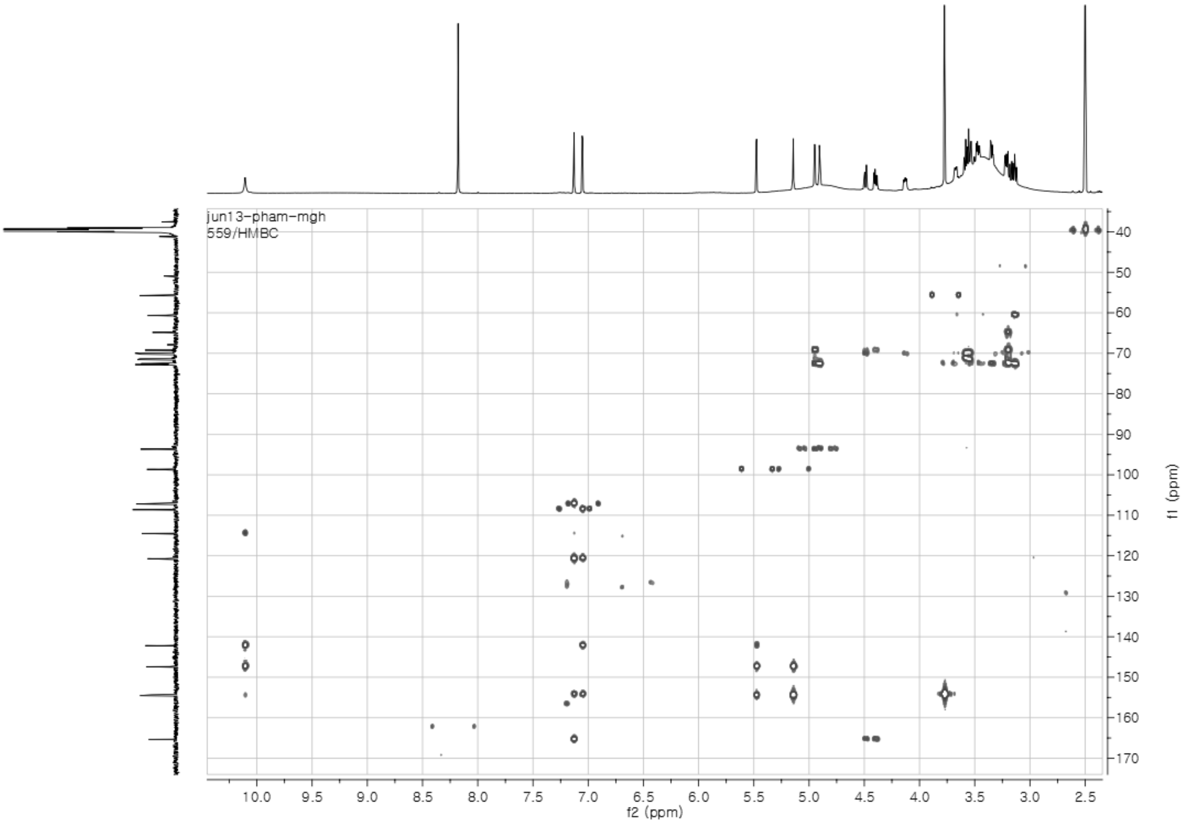

Figure S6. TOCSY spectrum (600 MHz) of Arcticoside (**1**) in DMSO-d_6.

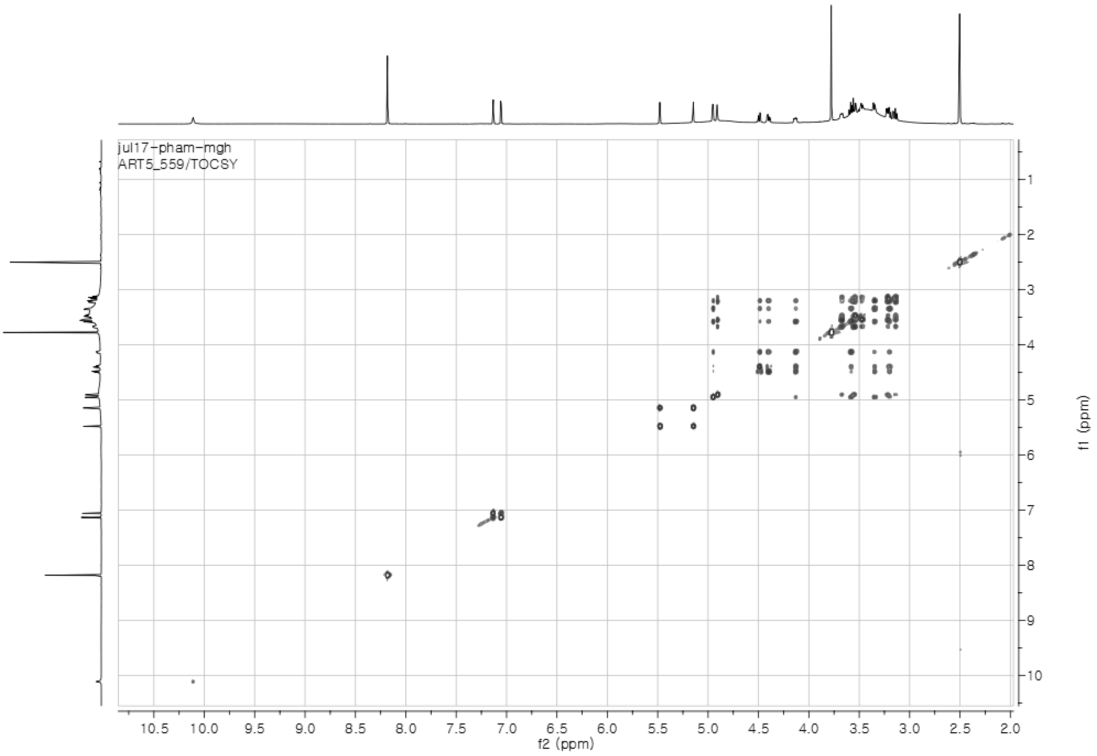

Figure S7. ROESY spectrum (600 MHz) of Arcticoside (**1**) in DMSO-d_6.

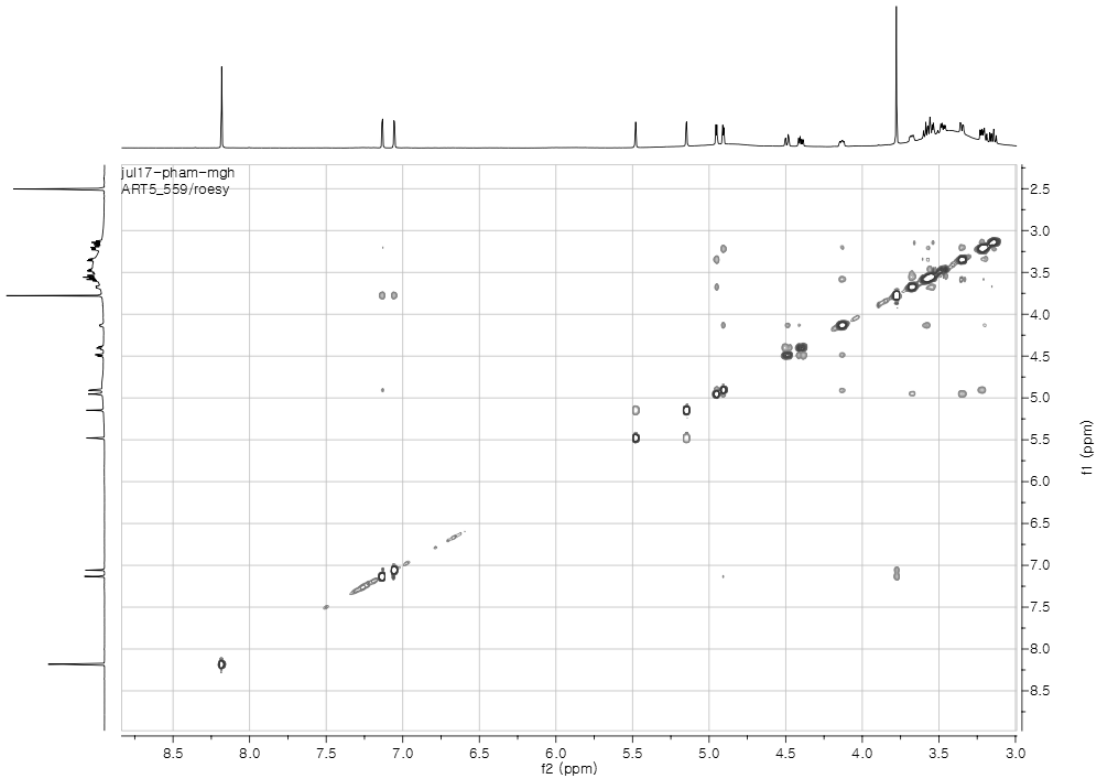

Figure S8. ^{1}H NMR spectrum (600 MHz) of C-1027 Chr-V (**2**) in DMSO-d_6.

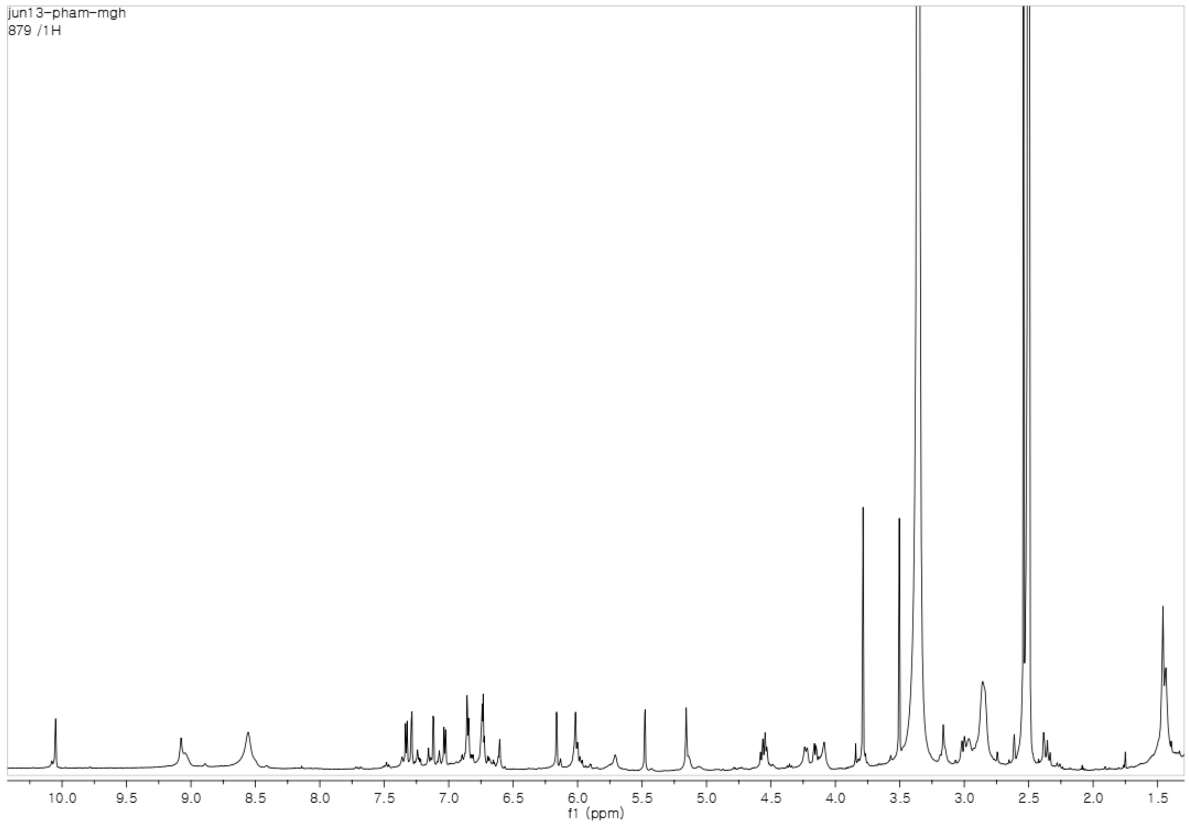

Figure S9. ^{13}C NMR spectrum (150 MHz) of C-1027 Chr-V (**2**) in DMSO-d_6.

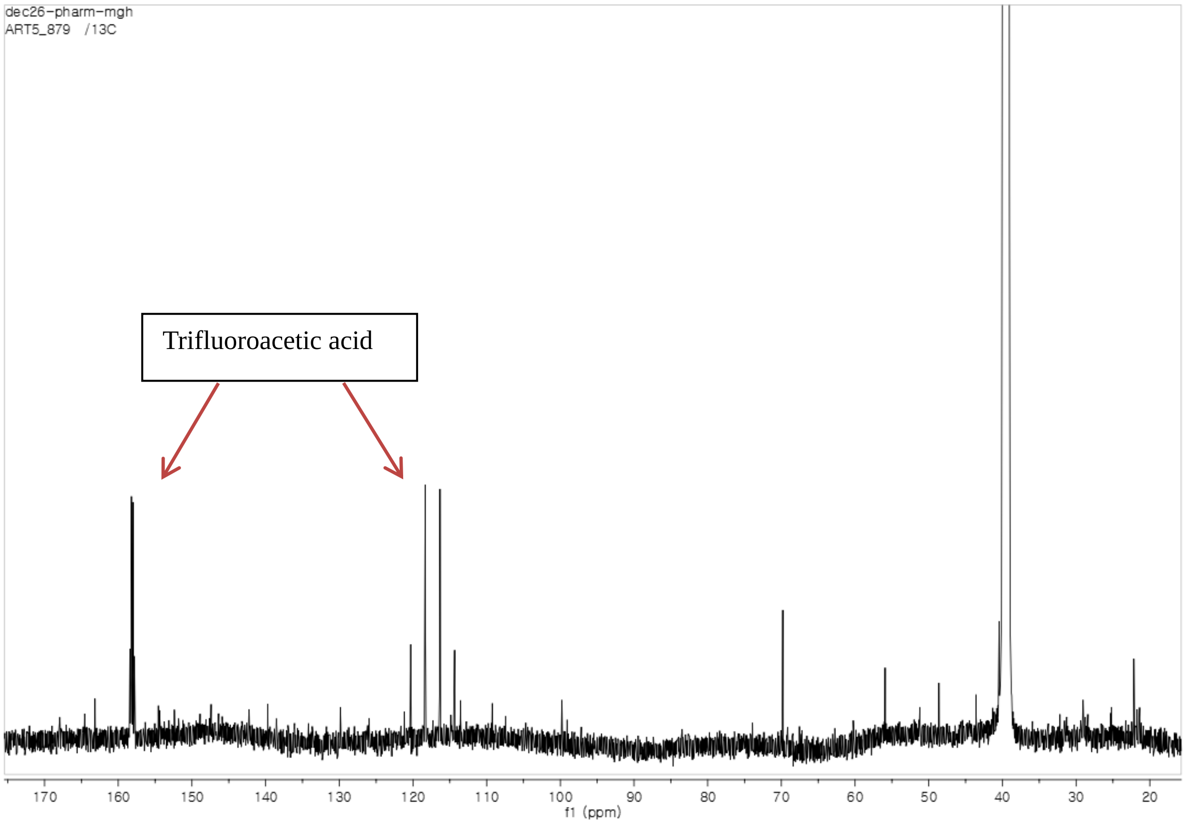

Figure S10. COSY spectrum (600 MHz) of C-1027 Chr-V (**2**) in DMSO-d_6.

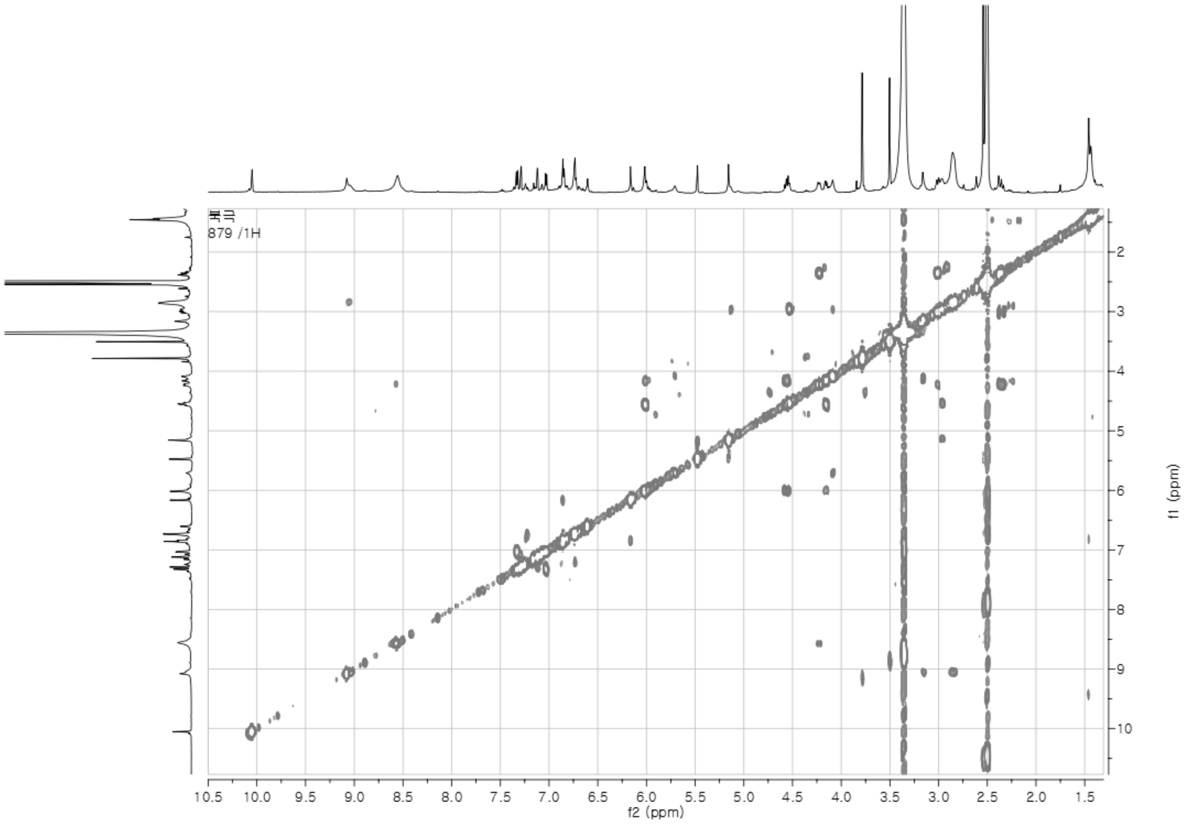

Figure S11. HSQC spectrum (600 MHz) of C-1027 Chr-V (**2**) in DMSO-d_6.

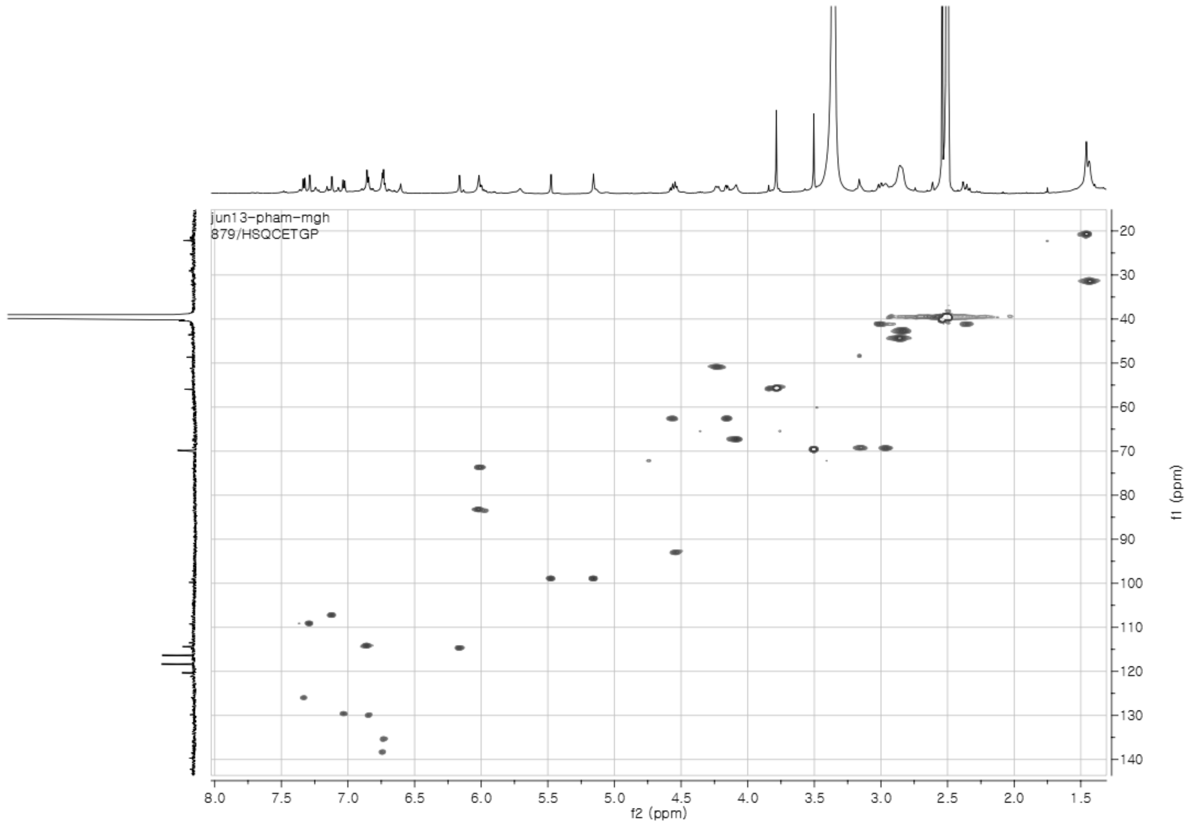

Figure S12. HMBC spectrum (600 MHz) of C-1027 Chr-V (**2**) in DMSO-d_6.

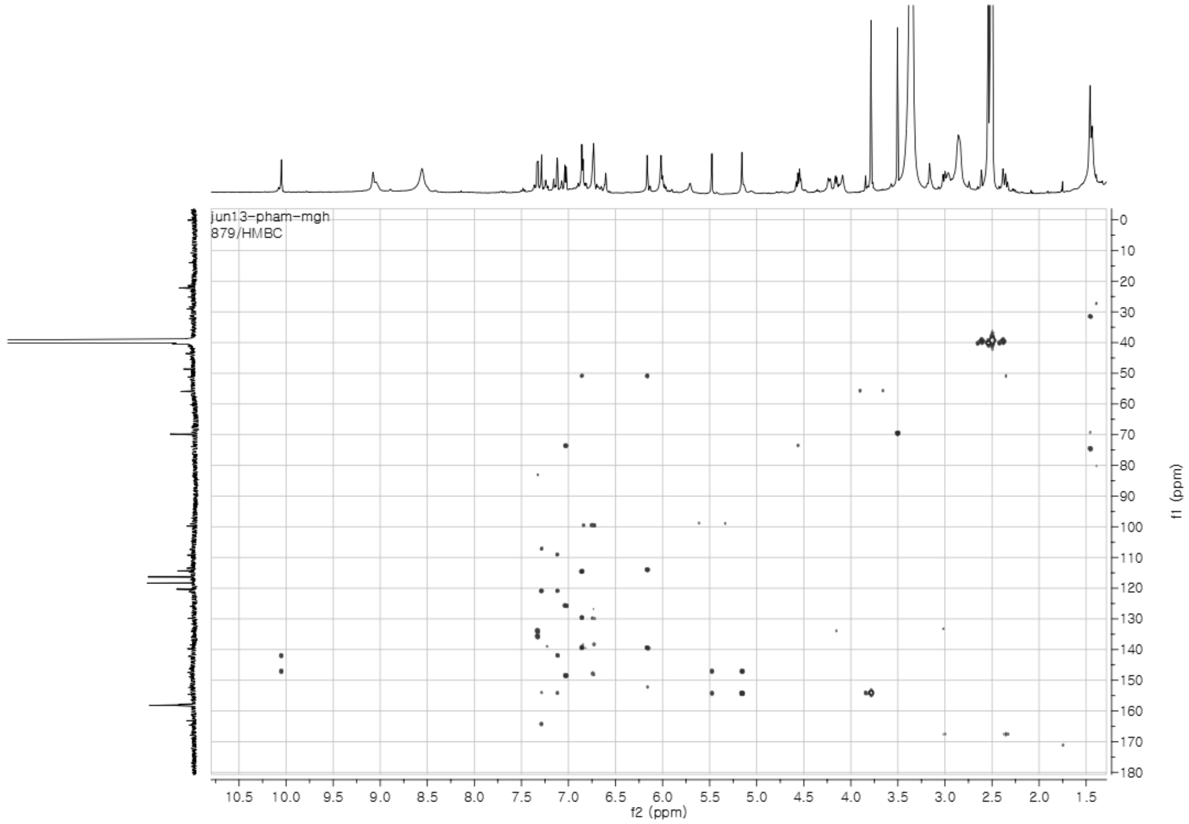

Figure S13. ROESY spectrum (600 MHz) of C-1027 Chr-V (**2**) in DMSO-d_6.

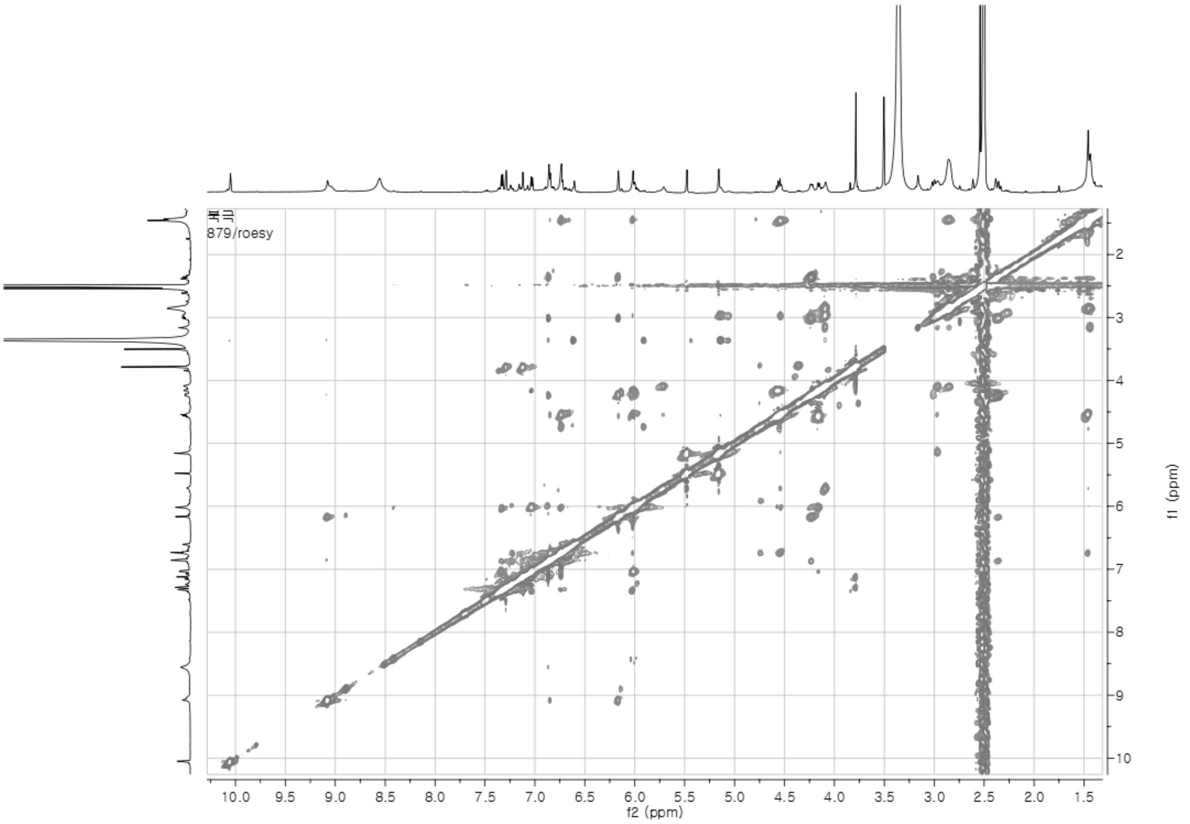

Figure S14. 16S rDNA sequence data of *Streptomyces* sp. ART 5.

CGCCTGGGCGGCGTGCTTTACCATGCAGTCGAACGATGAAATCACTTCGGTGG
TGGATTAGTGGCGAACGGGTGAGTAACACGTGGGCAATCTGCCCTTCACTCTG
GGACAAGCCCTGGAAACGGGGTCTAATACCGGATACCACTCTGTCCCGCATGG
GACGGGGTTGAAAGCTCCGGCGGTGAAGGATGAGCCCGCGGCCTATCAGCTTG
TTGGTGGGGTAATGGCCTACCAAGGCGACGACGGGTAGCCGGCCTGAGAGGG
CGACCGGCCACACTGGGACTGAGACACGGCCCAGACTCCTACGGGAGGCAGC
AGTGGGGAATATTGCACAATGGGCGAAAGCCTGATGCAGCGACGCCGCGTGA
GGGATGACGGCCTTCGGGTTGTAAACCTCTTTCAGCAGGGAAGAAGCGCAAGT
GACGGTACCTGCAGAAGAAGCGCCGGCTAACTACGTGCCAGCAGCCGCGGTA
ATACGTAGGGCGCAAGCGTTGTCCGGAATTATTGGGCGTAAAGAGCTCGTAGG
CGGCTTGTCACGTCGGATGTGAAAGCCCGGGGCTTAACCCCGGGTCTGCATTC
GATACGGGCTAGCTAGAGTGTGGTAGGGGAGATCGGAATTCCTGGTGTAGCGG
TGAAATGCGCAGATATCAGGAGGAACACCGGTGGCGAAGGCGGATCTCTGGG
CCATTACTGACGCTGAGGAGCGAAAGCGTGGGGAGCGAACAGGATTAGATAC
CCTGGTAGTCCACGCCGTAAACGTTGGGAACTAGGTGTTGGCGACATTCCACG
TCGTCGGTGCCGCAGCTAACGCATTAAGTTCCCCGCCTGGGGAGTACGGCCGC
AAGGCTAAAACTCAAAGGAATTGACGGGGGCCCGCACAAGCAGCGGAGCATG
TGGCTTAATTCGACGCAACGCGAAGAACCTTACCAAGGCTTGACATATACCGG
AAAGCATCAGAGATGGTGCCCCCCTTGTGGTCGGTATACAGGTGGTGCATGGC
TGTCGTCAGCTCGTGTCGTGAGATGTTGGGTTAAGTCCCGCA

Figure S15. (**a**) The GC chromatograms of the silylated arcticoside sugar. (**b**) Co-injection of the silylated D-glucose standard and the silylated arcticoside sugar. D-Glucose and the arcticoside sugar eluted at the same retention time. (**c**) Co-injection of the silylated L-glucose standard and the silylated arcticoside sugar. L-Glucose and the arcticoside sugar were separated.

(**a**)

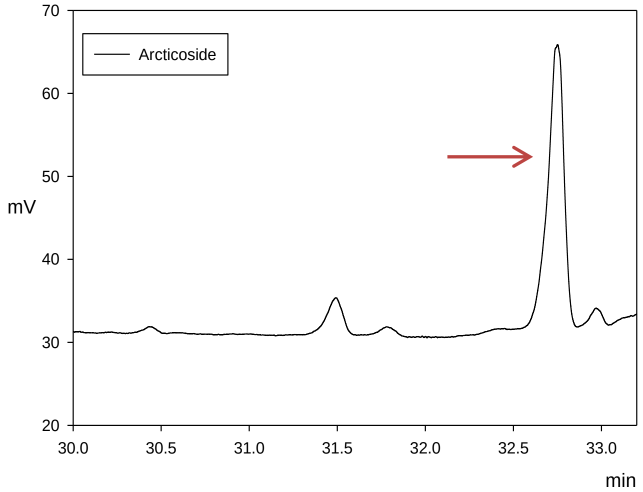

(**b**)

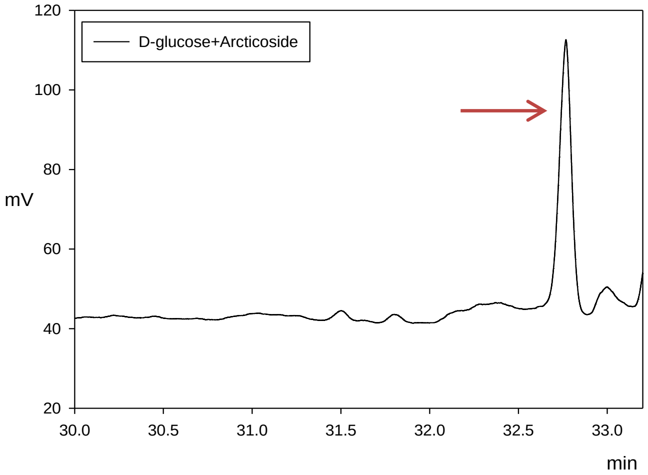

(**c**)

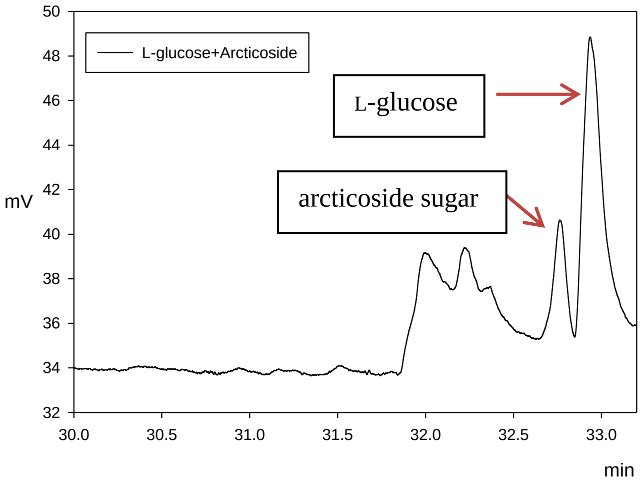

© 2014 by the authors; licensee MDPI, Basel, Switzerland. This article is an open access article distributed under the terms and conditions of the Creative Commons Attribution license (http://creativecommons.org/licenses/by/3.0/).
